# Nanomaterial-based strategies to overcome sorafenib resistance in hepatocellular carcinoma: from mechanistic insights to translational applications

**DOI:** 10.3389/fonc.2026.1839966

**Published:** 2026-05-20

**Authors:** Jinbang Huang, Shi Huang, Wenjing Chen, Shunsheng Wang, Zhihao Zuo, Xinyan Wu, Gang Deng

**Affiliations:** 1 Department of General Surgery, The Seventh Affiliated Hospital of Sun Yat-sen University, Shenzhen, China; 2 Clinical Medical College, Southwest Medical University, Luzhou, China; 3 The First Clinical College of Chongqing Medical University, Chongqing, China; 4 College of Food Science and Nutritional Engineering, China Agricultural University, Beijing, China

**Keywords:** clinical translation, hepatocellular carcinoma, nanomaterials, sorafenib resistance, targeted delivery

## Abstract

Hepatocellular carcinoma (HCC) is the most common histological subtype of primary liver cancer and a leading cause of cancer-related mortality. Although immunotherapy combinations have expanded systemic treatment options for advanced HCC, sorafenib remains clinically relevant in select patient populations and provides a mechanistically informative model for treatment resistance. Sorafenib resistance arises from interrelated processes, including insufficient intratumoral drug exposure, hypoxia-driven escape signaling, ABC transporter-mediated drug efflux, epithelial-mesenchymal transition, MAPK and PI3K/AKT/mTOR pathway compensation, ferroptosis dysregulation, and immunosuppressive microenvironment remodeling. Based on these mechanisms, we propose a mechanism-driven nanomedicine framework that integrates sorafenib delivery with targeted resistance-axis intervention, rather than focusing only on drug solubility, circulatory stability, or tumor accumulation. Representative strategies include ligand-targeted nanocarriers, CXCR4-directed delivery systems, the synchronous co-delivery of sorafenib with pathway inhibitors or nucleic acid regulators, ferroptosis-modulating nanodrugs, and tumor microenvironment (TME)-responsive delivery systems. Among these, biomimetic membrane-modified smart responsive platforms are particularly noteworthy because they can convert HCC microenvironmental features, such as elevated glutathione (GSH), immunosuppressive tumor-associated macrophage (TAM) accumulation, and ferroptosis resistance, into triggers for drug release, dual targeting, and resistance regulation. Artificial intelligence and machine learning may further support resistance-pattern prediction, patient stratification, nanoplatform selection, and formulation optimization. Overall, sorafenib nanomedicine may integrate drug delivery optimization, resistance intervention, and patient stratification into a unified therapeutic framework with improved mechanistic specificity and translational potential for sorafenib-resistant HCC.

## 1 Introduction

Primary liver cancer remains a major global health burden, with persistently high incidence and mortality rates worldwide (1). Hepatocellular carcinoma (HCC), the most common histological subtype, accounts for approximately 80% of primary liver cancers (2). Although systemic therapies have continued to evolve, durable clinical benefit in advanced HCC remains limited by drug resistance, insufficient tumor selectivity, and heterogeneous treatment response (3–5).

Sorafenib is a multi-kinase inhibitor that targets RAF-related signaling and VEGF/PDGF-mediated angiogenesis, and has long served as an important systemic treatment for advanced HCC (6, 7). However, its efficacy is frequently restricted by primary or acquired resistance. Sorafenib resistance is not driven by a single mechanism but involves interrelated processes, including ABC transporter-mediated drug efflux, epithelial-mesenchymal transition, hypoxia/HIF signaling, adaptive angiogenesis, compensatory bypass pathway activation, immune microenvironment remodeling, and impaired ferroptosis (6, 8, 9). These mechanisms collectively weaken the sustained antitumor activity of sorafenib and highlight the need for strategies that can address both drug exposure and resistance biology (6, 8).

**Figure 1:**
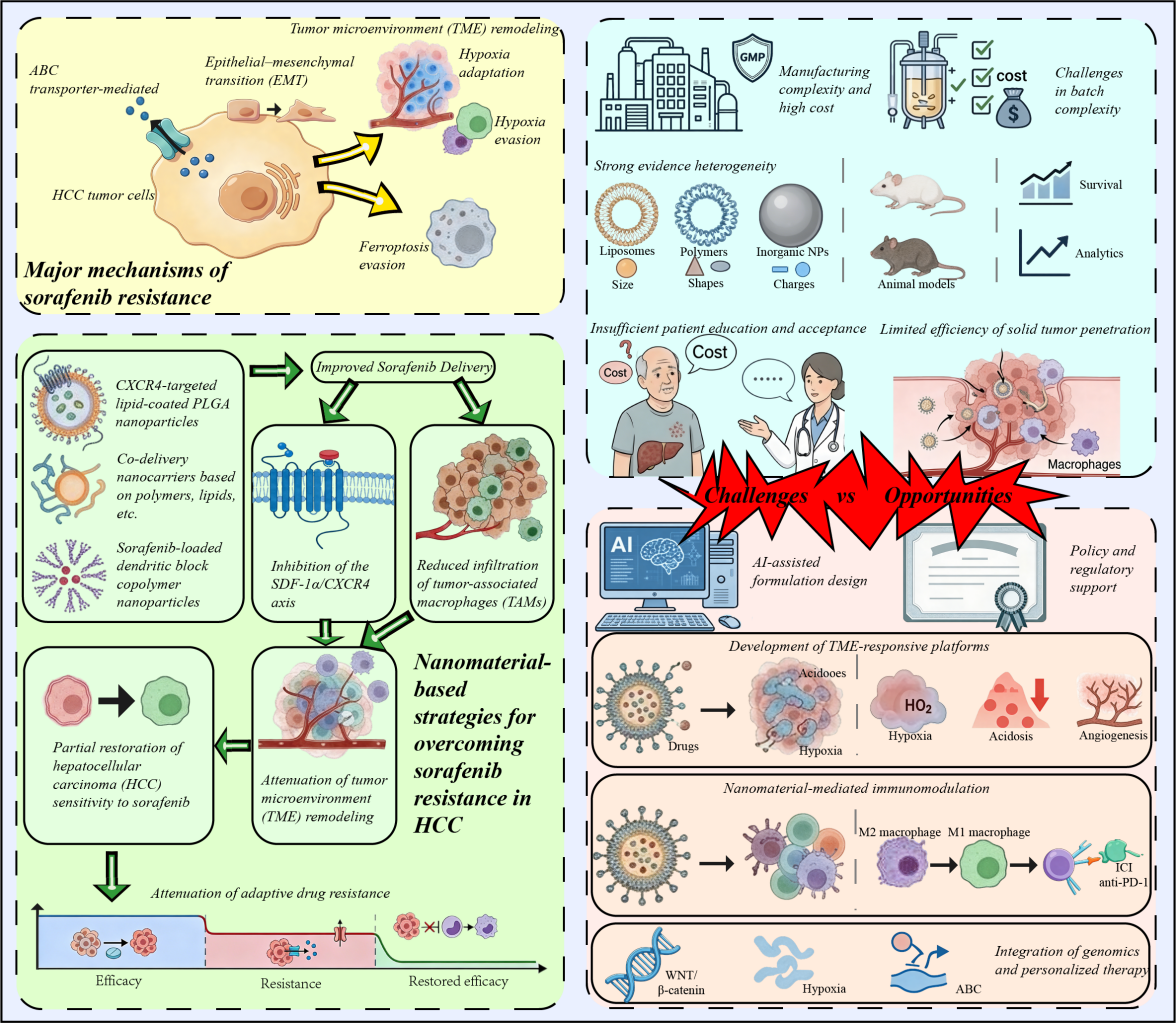
Schematic overview of the major mechanisms of sorafenib resistance in hepatocellular carcinoma, nanomaterial-based strategies to overcome resistance, and the key translational challenges and future opportunities in this field.

Nanomaterial-based delivery provides a rational approach to this problem. By improving the solubility, circulation stability, tumor accumulation, and intracellular retention of hydrophobic drugs such as sorafenib, nanocarriers may partially overcome pharmacokinetic and intratumoral delivery barriers (5, 10). More importantly, nanoplatforms can be engineered for targeted delivery and the co-delivery of resistance modulators and TME-responsive release (10, 11). In this Perspective article, we discuss sorafenib nanodelivery not merely as a formulation strategy but as a mechanism-guided platform for improving drug exposure and intervening in key resistance processes in HCC (**Figure 1**).

## 2 Why sorafenib nanodelivery remains valuable in the era of immunotherapy

As immunotherapy-based combinations increasingly reshape advanced HCC treatment, sorafenib-centered nanomedicine still requires justification (12–14). This focus does not represent a return to monotherapy but responds to persistent clinical and pharmacological realities.

First, sorafenib remains a drug suitable for nanomedicine optimization. (i) Sorafenib is limited by poor water solubility, insufficient effective tumor exposure, suboptimal systemic distribution, and dose-related adverse effects, all of which restrict its ability to maintain therapeutic concentrations within HCC lesions (8, 10). Nanodelivery platforms can potentially improve solubility, prolong circulation time, enhance tumor accumulation, and increase intracellular retention, thereby expanding the effective therapeutic window of sorafenib (5, 10). (ii) Sorafenib resistance is not solely driven by inadequate drug exposure but is also associated with hypoxia adaptation, SDF-1α/CXCR4 axis activation, drug efflux, bypass pathway compensation, immune microenvironment remodeling, and impaired ferroptosis (6, 8, 9). Therefore, controlled-release, targeted delivery, and co-delivery strategies may improve sorafenib exposure while simultaneously intervening in resistance-associated biological processes (10, 11).

Second, sorafenib has not been fully removed from the HCC treatment sequence. (i) For patients who are unsuitable for immune checkpoint inhibitor combinations or anti-angiogenic therapy because of autoimmune disease, long-term immunosuppression, organ transplantation, bleeding risk, untreated or high-risk gastroesophageal varices, severe cardiovascular comorbidities, or impaired hepatic reserve, sorafenib remains a feasible systemic option (14, 15). (ii) In many low- and middle-income countries, access to newer immunotherapy combinations is limited by drug costs, reimbursement restrictions, regulatory delays, and uneven healthcare infrastructure (16, 17). In these settings, sorafenib may remain the preferred or default targeted therapy when newer regimens are unavailable, unaffordable, or clinically unsuitable (4, 14).

Early clinical, controlled, and translational evidence also supports further evaluation of sorafenib nanodelivery. For example, lipid nanoparticle-loaded sorafenib combined with TACE after HCC resection was found to improve postoperative recurrence-free survival and overall survival compared with historical controls receiving transarterial chemoembolization (TACE) or sorafenib alone, with delayed recurrence and an approximately 60% 1-year survival rate (18). Although these findings remain preliminary, they suggest that nanoparticle-based sorafenib delivery could be useful in HCC settings where conventional treatment efficacy is limited or resistance is likely to emerge (10, 18).

## 3 Nanotherapeutic strategies: from drug delivery to reshaping the drug-resistance ecosystem

HCC lesions commonly exist within a microenvironment characterized by hypoxia, vascular abnormalities, stromal remodeling, immune suppression, and redox imbalance (6, 8, 9). These conditions restrict drug entry and intracellular retention while promoting bypass signaling, chemokine activation, immune remodeling, and ferroptosis resistance (6, 9). Therefore, nanomaterial-based therapy should move beyond generic carrier design toward mechanism-matched engineering. Different resistance processes require distinct nanosolutions.

### 3.1 Insufficient drug exposure and limited intracellular drug retention: shifting from “delivering more drug” to “maintaining effective intracellular exposure”

Sorafenib itself has poor water solubility, is rapidly cleared systemically, and has insufficient penetration into tumor tissue, making it difficult to maintain stable and adequate drug exposure within HCC lesions (10, 19). For this type of resistance, simply increasing the dosage does not effectively resolve the issue, as higher doses are often accompanied by more pronounced systemic toxicity, while local tumor drug concentrations and effective intracellular retention may still be insufficient (10, 19). Therefore, the significance of nanodelivery systems lies not merely in increasing the total dose of sorafenib delivered, but also in restructuring its *in vivo* distribution, tumor penetration, and intracellular release processes; this allows more sustained and effective drug exposure within resistant tumor cells (19, 20).

Liposomes, polymeric nanoparticles, dendrimers, and TPGS-modified nanosystems can overcome the hydrophobic limitations of sorafenib, increasing its apparent solubility and circulatory stability, thereby reducing the rapid clearance of the free drug and its non-specific distribution (19, 21). Through appropriate particle size control and surface modification, nanocarriers can also enhance local tumor accumulation and tumor cell uptake, enabling more of the drug to enter the interior of resistant cells (20). More importantly, strategies such as cancer cell membrane coating, creating platelet-like structures, or implementing other biomimetic membrane modifications can further enhance tumor homing, homophilic recognition, and circulatory stability (22, 23). This improves the selective accumulation of sorafenib within HCC lesions and provides a more favorable delivery foundation for subsequent intracellular release and drug retention. Additionally, certain nanoparticle platforms can release sorafenib in the TME or within endosomes and lysosomes via acid-, enzyme-, or redox-responsive mechanisms, bringing drug release closer to the intracellular site of action (11, 19). Enhanced cellular uptake and prolonged intracellular release also help reduce drug loss mediated by efflux, thereby mitigating drug resistance caused by insufficient drug exposure and limited intracellular retention (19, 20). For example, NP-TPGS-SFB was found to exhibit acid-responsive release, enhanced cellular uptake, and improved antitumor efficacy, which supports the use of material design as a strategy to improve exposure and intracellular retention (19).

For HCC patients with limited hepatic reserve, poor systemic tolerance, or dose-related adverse effects, improving tumor-selective exposure may be more feasible than increasing the sorafenib dose (7, 24). Future research should prioritize reproducible measurements of particle size, drug loading, release kinetics, liver clearance, and evidence of improved pharmacokinetic and safety profiles compared to free sorafenib.

### 3.2 Hypoxic adaptation and activation of the SDF-1α/CXCR4 axis: shifting from “anti-angiogenesis” to “blocking hypoxia-induced adaptive escape”

Sorafenib inhibits VEGF/PDGF-related angiogenesis. However, sustained anti-angiogenic pressure can induce hypoxia, activate HIF signaling and the SDF-1α/CXCR4 axis, and promote angiogenic escape and immunosuppressive infiltration (6, 25). Thus, increasing sorafenib delivery alone is insufficient; nanoplatforms should combine tumor delivery with blockade of hypoxia-driven adaptive escape (26).

The CXCR4-targeted nanosystem provides representative evidence for this approach (26). The AMD3100-modified CXCR4-targeted lipid-coated PLGA nanoparticles developed by Gao et al. not only improved sorafenib delivery efficiency in an HCC model but also utilized the CXCR4 antagonistic effect of AMD3100 to block the hypoxia-induced SDF-1α/CXCR4 axis, thereby reducing tumor-associated macrophage infiltration, inhibiting the progression and metastasis of *in situ* HCC, and prolonging the survival of tumor-bearing mice (26). The significance of this study lies in the fact that the nanoplatform is no longer merely a passive carrier for sorafenib but simultaneously performs drug delivery, chemotactic signal blockade, and regulation of the immune microenvironment (26). Similarly, LFC131-modified CXCR4-targeted PLGA-PEG nanoparticles that co-deliver sorafenib and metapristone were found to enhance cellular uptake, cytotoxicity, and apoptosis induction by targeting CXCR4-positive HCC cells, blocking SDF-1/CXCR4 interactions, and reducing CXCR4 expression (27). These studies demonstrate that nanostrategies targeting hypoxia-associated CXCR4 activation can simultaneously improve the efficiency of sorafenib delivery to tumors and intervene in hypoxia-induced chemotactic signaling and the remodeling of the immunosuppressive microenvironment.

Clinically, this strategy is most relevant for HCCs characterized by enhanced hypoxia, angiogenic escape, CXCR4 activation, or worsening immunosuppression after sorafenib exposure (25). Future studies should define patient-selection markers and validate whether CXCR4-targeted or hypoxia-modulating nanoplatforms can reproducibly improve tumor delivery, microenvironment regulation, and safety.

### 3.3 MAPK and PI3K/AKT/mTOR bypass activation: shifting from “monotherapy delivery” to “synchronized co-delivery and pathway blockade”

In addition to insufficient drug exposure, the compensatory bypass signaling formed by HCC cells under sorafenib treatment pressure is another key factor driving the continuous development of resistance (6). Long-term or low-dose sorafenib exposure can induce rebound activation of the MAPK pathway, promoting ERK activation, Bim downregulation, and enhanced cell survival; simultaneously, pathways such as PI3K/AKT/mTOR can serve as alternative survival signals, attenuating sorafenib’s antitumor effects (28–30). For this type of resistance, simply increasing the concentration of sorafenib often fails to resolve the issue, as tumor cells have already reestablished survival signals through these bypass pathways (6, 30). Therefore, a more rational strategy is to utilize a nano-co-delivery platform to simultaneously deliver sorafenib and bypass pathway inhibitors to the same tumor region or even within the same cell, ensuring better spatial distribution and temporal alignment between the two drug classes.

A nano-co-delivery system combining sorafenib with the PI3K/mTOR inhibitor BEZ235 provided more direct evidence for this strategy (30). This system primarily targeted compensatory PI3K/AKT/mTOR survival signals. By delivering sorafenib and BEZ235 simultaneously to resistant HCC cells, it enhanced the inhibition of cell proliferation and clonogenicity while promoting apoptosis, thereby improving the therapeutic efficacy of sorafenib against resistant cells (30). Thus, nanostrategies targeting bypassed signaling pathways should not be viewed merely as “vehicularized combination therapy,” but rather as a technique for spatially and temporally synchronized pathway blockade.

This approach is particularly relevant for HCC with PI3K/AKT/mTOR activation, MAPK rebound, or reduced sensitivity to sorafenib monotherapy (28–30). Future research should prioritize biomarker-based patient selection, optimized drug ratios, synchronized release kinetics, and toxicity management for combined pathway inhibition.

### 3.4 Immunosuppressive TME: shifting from “restoring sorafenib sensitivity” to “reshaping the immune response state”

Sorafenib resistance also occurs at the TME level (6). Hypoxia, TAM infiltration, PD-L1 upregulation, and impaired CD8^+^ T-cell function can form an immunosuppressive niche that reduces the response to sorafenib (31, 32). Therefore, nanomaterials should not only restore tumor-cell sensitivity but also modulate the immune state created after sorafenib treatment.

Nano-platforms can participate in this process through various mechanisms, such as regulating TAM infiltration, promoting antigen presentation, enhancing CD8^+^ T-cell infiltration, or co-delivering immunomodulators, thereby improving the local immune status following sorafenib treatment (32, 33). Among these, biomimetic membrane-modified platforms hold particular significance, as cancer cell membrane coating, platelet-like structures, and immune-related membrane components not only enhance tumor homing and local retention but also improve the adaptability of nanoparticles to the tumor immune microenvironment, making sorafenib delivery more easily combinable with TAM regulation, immune checkpoint blockade, or the restoration of T-cell function (23, 34). Studies of existing HCC models have shown that nanoplatforms that combine immune checkpoint inhibitors with sonodynamic therapy, biomimetic membrane modification, or metabolic regulation strategies can enhance CD8^+^ T-cell infiltration and deepen tumor regression, with superior efficacy compared to carrier-free delivery of the same drugs (23, 32, 33). These studies suggest that the value of nanomaterials lies not only in improving drug penetration into tumors but also in more closely integrating sorafenib therapy with antitumor immune activation through localized delivery and microenvironment regulation.

For HCC with TAM infiltration, PD-L1 upregulation, limited CD8^+^ T-cell infiltration, or Treg enrichment, immunomodulatory nanoplatforms may be more appropriate than delivery enhancement alone (35, 36). Biomimetic membrane-modified or dual-targeted systems are particularly attractive because they can improve tumor accumulation while delivering immunomodulatory modules to TAMs or other suppressive immune cells.

### 3.5 Ferroptosis resistance and adaptation to oxidative stress: shifting from “inducing cell death” to “overcoming anti-ferroptotic defenses”

Sorafenib can induce ferroptosis by affecting cysteine uptake, glutathione metabolism, and lipid peroxidation. However, HCC cells can also reduce their sensitivity to sorafenib through antioxidant and anti-ferroptotic mechanisms, such as NRF2, GPX4, and SLC7A11 (37–39). For this type of resistance, relying solely on sorafenib to induce oxidative damage is often insufficient to sustain effective cell death, as tumor cells have developed protective adaptations by enhancing antioxidant defenses, maintaining glutathione homeostasis, and inhibiting lipid peroxidation (37–39). Therefore, the focus of interventions for ferroptosis-related resistance should shift from merely “inducing damage” to “disabling anti-ferroptosis defenses.”

A nanodelivery system combining sorafenib with siNRF2 can silence NRF2, thereby weakening the antioxidant defenses of tumor cells and promoting the accumulation of lipid peroxides, thus restoring or enhancing sorafenib’s ability to induce ferroptosis (40). Furthermore, nanomaterials that are responsive to ROS, GSH, or an acidic microenvironment can enhance the selectivity of this strategy, allowing the carrier to remain relatively stable in normal tissues while triggering drug release and ferroptosis regulation within tumor cells characterized by redox imbalance, high GSH levels, or an acidic environment (11, 40). Thus, nanomaterials are no longer merely containers for delivering sorafenib but become engineered tools for regulating redox status and the mode of cell death.

These platforms are most relevant for HCC with enhanced antioxidant defenses or reduced ferroptosis sensitivity, such as tumors with elevated NRF2, GPX4, or SLC7A11 expression, active glutathione metabolism, or insufficient lipid peroxidation (37, 39). Future development should define ferroptosis-related biomarkers, optimize the ratio of sorafenib to redox- or gene-regulating modules, and balance ferroptosis enhancement with liver safety.

Overall, mechanism-specific nanosolutions redefine sorafenib delivery according to resistance biology (10). Nanoplatforms should maintain effective intracellular exposure, block hypoxia-driven escape, synchronize pathway inhibition, reshape antitumor immunity, and disable anti-ferroptotic defenses according to the dominant resistance axis (19, 23, 26, 30, 40). Through this mechanism-matched design, sorafenib nanodelivery can move beyond formulation optimization toward resistance-network intervention.

## 4 Smart stimulus-responsive nanomaterials: transforming the abnormal HCC microenvironment into therapeutic trigger signals

HCC lesions commonly exist within a microenvironment characterized by hypoxia, acidity, redox imbalance, elevated GSH levels, immunosuppressive TAM accumulation, and ferroptosis resistance (11, 41). These features not only contribute to sorafenib resistance but also provide endogenous signals for stimulus-responsive nanodelivery (11, 41). Compared with conventional responsive carriers that mainly rely on pH, ROS, or GSH changes for drug release, biomimetic membrane-modified platforms further exploit tumor cell membranes, platelet-like structures, and targeting peptides to enhance tumor homing, homologous recognition, and immune microenvironment adaptation (22, 23, 41). Thus, smart biomimetic sorafenib delivery can convert HCC microenvironmental abnormalities into targeting, release, and resistance-intervention signals.

Tang et al. developed a GSH-responsive biomimetic co-delivery platform using DS-PLGA as the responsive core to co-load sorafenib and an FSP1 inhibitor (41). Through HCC cell membrane coating and M2pep modification, this system achieved dual targeting of HCC cells and immunosuppressive M2-like TAMs (41). Elevated GSH levels in these cells triggered drug release, while FSP1 inhibition weakened anti-ferroptotic defenses and enhanced sorafenib-induced ferroptosis (41). This platform integrates sorafenib delivery, GSH-responsive release, ferroptosis sensitization, and TAM modulation, thereby transforming redox imbalance and TAM enrichment from resistance-driving factors into exploitable therapeutic triggers (41). Supporting this concept, Da et al. reported on biomimetic nanoplatelets co-delivering anti-PD-1 antibodies and sorafenib for HCC inhibition (23), while Lin et al. developed cancer cell membrane-coated PLGA nanoparticles co-loaded with sorafenib and superparamagnetic iron oxide nanoparticles (SPIONs) for integrated HCC therapy and imaging (22). Together, these studies indicate that biomimetic membrane-modified systems are not merely surface-engineered carriers but smart sorafenib delivery platforms capable of combining tumor targeting, responsive release, immune modulation, ferroptosis intervention, and theranostic monitoring (22, 23, 41).

Overall, biomimetic membrane-modified responsive platforms represent an important approach for exploiting the abnormal HCC microenvironment in sorafenib nanotherapy (22, 23, 41). Their translational value will depend on the reproducibility of the membrane source and coating, the controllability of the responsive release, the rationality of the co-delivery ratios, and liver safety.

## 5 AI/ML-assisted prediction of drug resistance patterns and nanocarrier optimization

With the increasing development of mechanism-specific nanoplatforms and stimulus-responsive materials, the key challenge in treating sorafenib-resistant HCC is no longer simply whether effective carriers can be constructed, but rather how to select the most appropriate platform for a given patient and optimize its parameters (10, 11). HCC exhibits substantial molecular and spatial heterogeneity, and different tumors may be dominated by distinct resistance patterns, including hypoxia adaptation, SDF-1α/CXCR4 activation, bypass signaling, drug efflux, immune suppression, or ferroptosis resistance (6, 8). Therefore, nanosorafenib therapy should move beyond a one-size-fits-all design toward resistance-informed platform selection. AI/ML provides a practical framework to connect resistance prediction, patient stratification, nanoplatform selection, and formulation optimization (42–44).

Regarding resistance-pattern prediction, machine learning has been applied to HCC prognostic stratification, sorafenib response prediction, and the identification of resistance-associated cell states (42, 45). Wu et al. integrated The Cancer Genome Atlas (TCGA), International Cancer Genome Consortium (ICGC), Gene Expression Omnibus (GEO), and single-cell sequencing data to establish immune developmental-related features for predicting HCC prognosis and sorafenib resistance (42). Importantly, this study further identified regulatory T cell (Treg) subsets associated with sorafenib resistance and the potential biomarker basic leucine zipper ATF-like transcription factor (BATF), extending prediction from bulk transcriptomic scores to specific immune cell states and candidate molecular markers (42). Luo et al. also constructed a sorafenib resistance-related gene signature and showed that resistance status is closely associated with immune infiltration patterns and TME remodeling (45). These studies suggest that AI/ML should not be limited to predicting prognosis or drug response, but should help identify clinically actionable resistance patterns (42, 45). To be translated into clinical practice, however, these models need to be integrated with clinical features, liver function, radiomics, pathology, immune infiltration assessment, and selected molecular biomarkers to support feasible patient stratification.

In nanocarrier optimization, AI/ML can convert empirical formulation screening into a predictable and iterative design process (43, 44). Particle size, surface charge, material composition, ligand density, drug-to-carrier ratio, membrane modification, and release profile all influence tumor accumulation, tissue distribution, cellular uptake, hepatic clearance, toxicity, and therapeutic response (43, 44). Lin et al. used the Nano-Tumor Database and PBPK modeling data to predict nanoparticle tumor delivery efficiency, transforming delivery efficiency into a quantifiable and optimizable parameter (43). Chou et al. further developed an AI-assisted physiologically based pharmacokinetic (PBPK) model to predict nanoparticle delivery efficiency and *in vivo* distribution in mouse tumors based on physicochemical properties, supporting early screening of candidate nanomedicines (46). More recently, Chan et al. proposed the COMET model, which used a large lipid nanoparticle dataset to train a Transformer neural network that integrates lipid structures, component ratios, and preparation parameters to predict lipid nanoparticle (LNP) performance and virtually screen candidate formulations (44). This study established a methodological precedent for using deep learning to navigate high-dimensional nanocarrier formulation spaces, offering a transferable strategy for the future optimization of sorafenib-loaded nanoparticles with defined delivery, stability, and release characteristics (44).

For sorafenib nanodelivery, similar models could incorporate particle size, drug loading, surface ligands, biomimetic membrane modification, responsive release, hepatic uptake, tumor accumulation, toxicity, and efficacy to identify candidate formulations suited to different HCC resistance patterns (43, 44, 46). In particular, for biomimetic membrane-modified or stimulus-responsive sorafenib platforms, AI/ML can help determine if the membrane source, ligand density, co-delivery ratio, and release kinetics match specific HCC microenvironmental features (41, 44). Thus, AI/ML can establish a continuous translational pathway from resistance prediction to platform selection and AI-assisted formulation design, moving sorafenib nanodelivery from empirical material optimization toward data-driven precision nanomedicine.

## 6 Conclusion

Sorafenib nanomedicines should not be viewed as merely repackaging an older targeted drug; rather, they should be seen as a mechanism-guided strategy for addressing persistent barriers in HCC treatment. Their value lies in linking sorafenib delivery with resistance intervention, including enhanced tumor exposure, co-delivery of resistance modulators, microenvironment-responsive release, and immune- or ferroptosis-related remodeling. Among emerging research directions, biomimetic membrane-modified responsive platforms and AI/machine learning-aided optimization are particularly important, as they may enable more precise tumor targeting, platform selection based on resistance patterns, and rational formulation design. In terms of clinical translation, future research should move beyond the proof-of-concept stage to establish standardized manufacturing processes, reproducible pharmacokinetic data, liver safety assessments, biomarker-based patient screening, and prospective validation. Through these initiatives, sorafenib nanodelivery technology is expected to evolve from an experimental formulation improvement into a clinically valuable precision nanomedicine strategy for treating sorafenib-resistant hepatocellular carcinoma.

## Data Availability

The original contributions presented in the study are included in the article/supplementary material. Further inquiries can be directed to the corresponding authors.
